# The Effect of Breakfast Prior to Morning Exercise on Cognitive Performance, Mood and Appetite Later in the Day in Habitually Active Women

**DOI:** 10.3390/nu7075250

**Published:** 2015-07-14

**Authors:** Rachel C. Veasey, Crystal F. Haskell-Ramsay, David O. Kennedy, Brian Tiplady, Emma J. Stevenson

**Affiliations:** Brain, Performance and Nutrition Research Centre, Faculty of Health and Life Sciences, Northumbria University, Newcastle upon Tyne NE18ST, UK; E-Mails: crystal.haskell-ramsay@northumbria.ac.uk (C.F.H.-R.); david.kennedy@northumbria.ac.uk (D.O.K.); brian@penscreen.com (B.T.); e.stevenson@northumbria.ac.uk (E.J.S.)

**Keywords:** breakfast, exercise, cognition, mood, females

## Abstract

Pre-exercise nutritional practices for active females exercising for mood, cognitive and appetite benefits are not well established. Results from an initial field pilot study showed that higher energy intake at breakfast was associated with lower fatigue and higher overall mood and alertness post-exercise (all *p* < 0.05). In a follow-up, randomised, controlled trial, 24 active women completed three trials in a balanced, cross-over design. At 0815 h participants completed baseline cognitive tasks, mood and appetite visual analogue scales (VAS) and were administered a cereal breakfast (providing 118 or 236 kcal) or no breakfast. After 45 min, they completed a 30 min run at 65% heart rate reserve (HRR). Parameters were re-assessed immediately after exercise, then hourly until lunch (~1240 h), immediately post-lunch and at 1500 and 1900 h via a mobile phone. Breakfast enhanced feelings of relaxation before lunch (*p* < 0.05, *d* > 0.40), though breakfast was detrimental for working memory mid-afternoon (*p* = 0.019, *d* = 0.37) and mental fatigue and tension later in the day (all *p* < 0.05, *d* > 0.038). Breakfast was also beneficial for appetite control before lunch irrespective of size (all *p* < 0.05, *d* > 0.43). These data provide information on pre-exercise nutritional practices for active females and suggest that a small breakfast eaten prior to exercise can benefit post-exercise mood and subjective appetite ratings.

## 1. Introduction

Many people exercise regularly for physical and psychological benefits, which are well documented in the literature (e.g., [1,2]). Acutely, exercise can improve cognitive function [3], psychological state and mood [4,5] and support weight regulation [1]. These variables can also be positively influenced by following healthy dietary practices and it is widely believed that one such practice is the regular consumption of breakfast [6,7,8]. Breakfast is a likely source of pre-exercise nutrition if exercise is undertaken in the morning but is a frequently omitted meal among young females [9,10]. Pilot survey data in an active female population revealed that the main reasons for skipping breakfast before morning exercise were lack of time and avoiding discomfort during exercise (unpublished data), which mirrors findings of a previous study in swimmers [11]. In addition, a common reason for females to exercise is for weight management [9,12], and as exercise in a fasted state can increase fat oxidation [13,14], they may also follow this practice to maximize their weight loss potential.

Little is known about how pre-exercise nutrition affects post-exercise cognitive performance, mood and appetite. Specifically, the relationship between breakfast consumption and exercise and the subsequent effects on these parameters has not been explored in depth and preliminary research in this area has yielded conflicting results. In active adult males, breakfast consumption (451 kcals), compared to omission, prior to exercise has been found to reduce mental fatigue [15] and reduce subjective appetite [13] post-exercise. Conversely, other studies have shown no effect of breakfast consumption prior to exercise on cognitive performance [15,16] (451 kcals and 281 kcals, respectively) or mood [11,16] (451 kcals and 200 kcals, respectively). Although there are nutritional guidelines available for athletes to follow with regards to exercise performance, for example, consuming carbohydrate (CHO) following an overnight fast and 2–4 h before exercise [17], these guidelines may not be applicable for those exercising recreationally rather than for competition or performance enhancement. In a recent survey only 23% of individuals (*n* = 699) who regularly engaged in moderate intensity physical activity reported that they preferred not to have breakfast before exercise. At least 40% of the sample agreed that they had more energy on days that they ate breakfast, that eating breakfast led to increased physical activity, and that they felt they must eat breakfast before they engaged in exercise [18].

It has been suggested that women are more sensitive to changes in mood and appetite than men [19,20], and therefore may be more susceptible to any negative effects of omitting breakfast, particularly prior to exercise. To date, no studies have assessed the effect of consuming a typical breakfast prior to exercise on post-exercise cognitive performance, mood, appetite and energy intake (EI) in recreationally active females; the current paper describes two studies which aimed to explore this paradigm. To begin, a pilot field study was conducted to investigate whether consuming breakfast prior to morning exercise affects cognitive performance, mood, appetite and energy intake (EI) for the remainder of the day. The results from this study suggested that breakfast size may influence post-exercise mood and appetite. Subsequently, a randomized controlled trial was conducted to see if a small breakfast, which was quick to consume and digest and therefore perhaps be suited to this population, could lead to cognitive or mood benefits over no breakfast or consuming a larger breakfast prior to a morning exercise session; this study will provide the main focus of the paper.

## 2. Experimental Section

### 2.1. Pilot Study

#### 2.1.1. Methods

A preliminary, single-day field study was conducted to assess whether energy intake at breakfast prior to morning exercise influenced cognitive function, mood, appetite and energy intake (EI) for the remainder of the day.

##### Participants

Forty five, healthy, habitually active females (defined as exercising for at least 30 min, 3 times per week for at least the previous 6 months; [21]) were recruited and completed the study. Their mean ± SD age, height, body mass (BM) and body mass index (BMI) score were 21.4 ± 3.3 years, 168.6 ± 0.1 cm, 62.4 ± 7.8 kg and 21.8 ± 2.8 kg/m^2^ respectively. As well as being habitually active and regularly exercising in the morning, participants also confirmed they were aged 18–35 years, in good health, a non-smoker, free from medication and herbal and dietary supplements and had no history of head trauma, learning difficulties, attention deficit hyperactivity disorder, dyslexia, migraines or gastric problems, a good standard of English and a healthy BMI (>18 and <25 kg/m^2^) and blood pressure (≤140/90 mmHg).

##### Screening and Familiarisation

Ethical approval for this study was granted by the Ethics Committee of the Faculty of Health and Life Sciences at Northumbria University and was conducted according to the Declaration of Helsinki. Prior to participation volunteers gave written informed consent. They were then familiarised with the mobile phone cognitive tasks, mood and physical state visual analogue scales (VAS; taken from Rogers *et al.*, 2003 [22]) and appetite VAS (for full descriptions of the mobile phone tasks and VAS see Document S1), completing them 3 times to reduce learning effects. They also received detailed instruction on recording food intake using a food diary and completed the Three-Factor Eating Questionnaire (TFEQ) R-18, a measure of dietary restraint [23]; mean ± SD TFEQ score for the sample was 21.6 ± 5.9.

##### Experimental Procedures

Each participant completed a single study day which took place in their own environment where they undertook a morning exercise session (between the hours of 0600 and 1100 h) which was of typical mode and intensity for them. Details of this exercise session (time, type, duration, intensity) were recorded in a log immediately after the session. They also completed the mobile phone cognitive tasks upon waking (baseline), at 1130 h, 1500 h and 2000 h. Food intake and physical activity were recorded from breakfast the day preceding, until breakfast the day after, the study day.

#### 2.1.2. Statistic

Food diaries were analysed using Microdiet (Downlee Systems Ltd, Chapel-en-le-Frith, Derbyshire, UK). Scores for each individual task were analysed as “change from baseline” using SPSS 19 (SPSS, Inc., Chicago, IL, USA) and averaged across the 3 post-exercise time points to give overall scores for each variable. Partial correlations were conducted to assess the effect of the size of breakfast consumed prior to exercise on post-exercise scores. Breakfast was defined as anything other than waterconsumed between waking and exercise; non-breakfast consumers were included in the analysis. Ratings for exercise intensity (mm) were multiplied by exercise duration (min) to give each individual an overall exercise workload score which was used as a covariate in all analyses. An alpha level of 0.05 was used for all statistical tests. Significant results are reported. Confidence intervals are reported in square brackets and means and Standard Error Means (SEM) are reported where appropriate.

### 2.2. Main Study

#### 2.2.1. Methods

A randomized, balanced, cross-over study was conducted to compare the effects of two different sized breakfasts and no breakfast consumed prior to morning exercise on cognitive function, mood, appetite and energy intake (EI) for the remainder of the day.

##### Participants

Twenty five young, healthy, habitually active females took part in the study. One participant withdrew due to time commitments, leaving a final sample of 24. Their mean ± SD age, height, body mass (BM) and Body Mass Index (BMI) were 20.9 ± 2.3 years, 170.0 ± 7.0 cm, 63.0 ± 6.4 kg, 21.9 ± 1.9 kg/m^2^ respectively. In addition to the general inclusion criteria listed for the pilot study (see Section 2.1.1), participants also confirmed they were habitually active (exercising for at least 30 min, 3 times per week for at least the previous 6 months; [21]), regularly exercised in the morning, were able to run for 30 min continuously at a moderate speed, habitually consumed breakfast prior to a morning exercise session and had a liking for all of the food items provided in the study. Restrained eating was measured at screening using the TFEQ R-18 [23] but not used as an inclusion criterion. All participants but one were restrained eaters, scoring >10 on the TFEQ. Where participants were not using hormonal contraception, both main trials were conducted during the same phase (either luteal or follicular) of their menstrual cycle where possible.

##### Screening and Familiarisation

Ethical approval for this study was granted by the Ethics Committee of the Faculty of Health and Life Sciences at Northumbria University and was conducted according to the Declaration of Helsinki. Prior to participation volunteers gave written informed consent. Participants completed a screening session to assess eligibility and demographic, anthropometric and blood pressure measures were collected. Familiarisation with the experimental procedures and the computer and mobile phone cognitive tasks and VAS was provided. Participants completed the computerized and mobile phone tasks 3 times before taking part in the study to reduce the chance of learning effects on main trial days. Participants were also fully briefed on how to correctly complete a food diary and were provided with a set of electronic kitchen scales where necessary.

##### Cognitive Tasks

The computerized cognitive tasks were delivered via the Computerised Mental Performance Assessment System (COMPASS, Northumbria University), a programme used to present standard psychometric tests. COMPASS has been used in several previous nutritional intervention studies and has been shown to be sensitive to cognitive enhancement following a variety of nutritional interventions [24,25,26]. The COMPASS tasks comprised of Four Choice Reaction Time, Stroop, N-Back and a Rapid Visual Information Processing task (for full task descriptions see Document S1). Data from studies measuring episodic memory measured using a word recall task provide the most convincing positive evidence for the effect of breakfast on cognitive performance. However, more research is needed focusing on other cognitive domains and tasks before concrete conclusions can be drawn. Furthermore, it has been suggested that null findings in studies investigating the effect of breakfast on cognitive function may be due a lack of sensitivity in tasks measuring cognitive domains other than memory [27]. This advocates further exploration using different tasks, perhaps steering away from the very commonly used word recall task.

The mobile phone cognitive tasks consisted of an N-Back task (as used in Kennedy *et al.*, 2011 [28]), an RVIP task and an Arrow Reaction Time task (for full mobile phone task descriptions see Document S1).

##### Visual Analogue Scales (VAS)

Prior to cognitive task completion, VAS were used to measure mood and physical state (“relaxed”, “alert”, “jittery”, “tired”, “tense”, “headache”, “overall mood”), mental fatigue and task difficulty, breakfast and lunch “liking” and exercise enjoyment. Subjective appetite ratings (“hunger”, “fullness”, “desire to eat” and “satisfaction” were also recorded using VAS, a valid and sensitive method of measuring appetite [29]. All VAS were completed electronically with the exception of the appetite VAS completed during exercise which were pen and paper (for full VAS descriptions see Document S1).

##### Preliminary Exercise Tests

Participants also undertook two preliminary exercise tests. The first established the relationship between heart rate (HR) and running speed on a flat treadmill using a 16 min test. The test began at a low-moderate speed (between 7–8.5 km·h^−1^). Every 4 min, the speed of the treadmill was increased by 1 km·h^−1^ until 16 min had elapsed. HR was recorded during the last minute of each stage. Participants were allowed 5–10 min recovery after the preliminary test before undertaking a maximal exercise test to establish their maximum HR (HR_max_). An incremental treadmill test was utilized [30] whereby the speed of the treadmill remained constant (typically 9–11 km·h^−1^) but the gradient of the treadmill was increased by 1%·min^−1^ to exhaustion. Verbal encouragement was given towards the latter stages of the test to ensure that subjects worked to exhaustion*.* Reaching a HR within 10 beats·min^−1^ of age-predicted HR_max_ was deemed satisfactory. The running speed equivalent to 65% of each participant’s heart rate reserve (HRR) was then determined. HRR is accepted as an accurate method of controlling exercise intensity [31] and is calculated as HR_max_—resting HR. Previously, 60%–70% of HRR has been used to achieve exercise of moderate intensity [32].

##### Pre-Testing Procedures

On the day preceding the first trial, participants kept a record of their food intake and physical activity. They were required to replicate these behaviours and diet the day preceding subsequent trials. In the 24 h period prior to each main trial, participants abstained from alcohol, caffeine and vigorous physical activity.

Participants consumed a standardized meal the evening prior to each main trial which was provided by the researcher, as cognitive responses to breakfast can be influenced by food consumed the previous evening [33]. This meal consisted of a pack of Uncle Ben’s grilled Mediterranean vegetable microwave risotto (412 kcal, 78.8 g CHO, 9.6 g protein, 6 g fat) prepared with water as per the manufacturer’s instructions and an Ambrosia low-fat custard pot (134 kcal, 23.2 g CHO, 4.4 g protein, 2.7 g fat). Participants were required to consume this meal before 2000 h the evening before each main trial, and ensure this was the last food they consumed before each of the test sessions. They were also required to fast for 12 h prior to the start of each main trial.

##### Treatments/Test Meals

The nutritional content of the breakfast and lunch provided in the study are detailed in Table 1. In the two breakfast trials, participants were given a breakfast consisting 40 g or 20 g of Special K breakfast cereal (Kellogg’s, Manchester, UK) with 166 mL and 83 mL of semi-skimmed milk (Sainsbury’s, London, UK) respectively. These amounts were chosen based on the manufacturer’s recommendation of 30 g of cereal, which was considered to be an average serving. Participants were given a maximum of 10 min to consume the breakfast. Each portion of the *ad libitum* lunch was prepared in advance and re-heated in the microwave for 5 min as required. Participants were initially served 400 g of pasta. Approximately every 3–5 min, more pasta was added to the bowl (300 g initially, then 100 g thereafter) until the participant indicated that they were full. Participants were not allowed to completely finish a portion before the bowl was refilled.

**Table 1:** Nutritional content of study foods.

	kJ/Kcal	Carbohydrate (g)	Protein (g)	Fat (g)	Fibre (g)
Breakfasts					
20 g Special K cereal	321/76	15	3	0.3	0.9
83 mL Semi-skimmed milk	171/42	4	1.5	1.5	<0.5
Total	492/118	19	4.5	1.8	0.9
40 g Special K cereal	642/152	30	6	0.6	1.8
166 mL Semi-skimmed milk	342/81	8	3	3	<0.5
Total	984/236	38	9	3.6	1.8
Lunch					
125 g Penne Pasta	1894/446	91	15	2	3
250 g Tomato and Herb pasta sauce	540/128	20	4	3	4
15 g Olive Oil	508/123	<0.5	<0.5	14	<0.5
40 g Cheddar Cheese	648/156	<0.5	10	13	<0.5
Total per 430 g portion	3544/938	111	29	32	7
Total per 100 g	824/218	26	7	7	1.5

##### Experimental procedures (Figure 1)

Each participant completed three trials, in a randomised, cross-over design, where they were administered one of three breakfasts: 40 g cereal + 166 mL milk, 20 g cereal + 83 mL milk or no breakfast. The first trial was undertaken between 48 h and 14 days of the initial screening visit. Trials were separated by ≥48 h and all trials were performed under similar laboratory conditions. Trials began at 0815 h (±15 min). After confirming compliance to the study restrictions, participants completed baseline cognitive tasks and mood and appetite VAS before being administered the test breakfast or remaining fasted. Participants were then required to rest for 45 min to allow for the digestion of breakfast before repeating the cognitive tasks and mood and appetite VAS. Immediately following this, they undertook a 30 min treadmill run (at approximately 65% HRR). Heart rate was monitored throughout the exercise period using telemetry (Polar T31 transmitter, Polar Electro Oy, HQ, Professorintie 5, FIN-90440 Kempele, Finland) and Rate Perceived Exertion (RPE [34]) was measured at 5 min intervals. Participants then completed the cognitive tasks and mood and appetite VAS immediately after, and at 1 and 2 h, post-exercise. They were then administered an *ad libitum* pasta lunch where they were asked to consume enough food to feel satisfied to a normal level. After lunch, they completed the cognitive battery and mood and appetite VAS for a final time and were then free to leave the laboratory. Water intake was recorded during the first trial and was matched in the subsequent trials. Participants also completed similar cognitive tasks, mood and appetite VAS via a mobile phone at 1500 and 1900 h and completed a food diary for the rest of the day.

**Figure 1:**
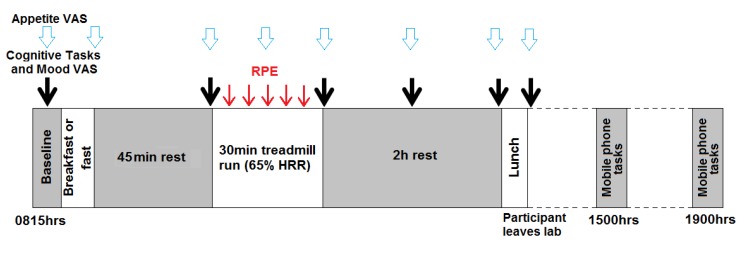
Main study schematic.

#### 2.2.2. Statistical Analysis

A previous study investigating the effect of breakfast prior to exercise on mood, cognitive function and appetite at 4 time-points post-exercise yielded medium-large effects (Cohen’s D (*d*) = 0.5–0.8). Based on this, a power calculation (based on number of cognitive task repetitions) carried out *a priori* indicated that a total sample size of 24 would provide statistical power to detect large effects above 80% with an alpha level of 0.05 [35,36].

Before the main statistical analysis, a one-way ANOVA was used to compare pre-breakfast baseline data to assess for differences in performance across the main trials. Scores for each individual task outcome completed on the computer (MPSVAS, FCRT, Stroop, N-back, RVIP, mental fatigue VAS and task difficulty VAS) were analysed as “change from baseline”. All appetite VAS were analysed as time-averaged AUC, calculated using the trapezoidal method. Data for the breakfast and lunch liking VAS, exercise enjoyment VAS, RPE, EI and the mobile phone tasks were analysed as absolute values. TFEQ scores were used as a covariate in the EI analysis only as it has been previous found that restraint scores calculated using the TFEQ were not associated with appetite sensations [37]. Data were split into four parts for analysis: pre-exercise (not presented in this paper), pre-during-post exercise (appetite only), post-exercise and mobile phone data. A 3 × 4 (breakfast × repetition) repeated-measures ANOVA was used to assess differences in performance and mood during the post-exercise time-points in the laboratory and a 3 × 2 (breakfast × repetition) ANOVA for the mobile phone data analysis. A one-way repeated-measures ANOVA was used when data was collected only at a single time point (for example, pre-exercise). All analyses were carried out using SPSS 19 (SPSS, Inc., Chicago, IL, USA). Planned comparisons (using *t* tests calculated with the Mean Squares Error) were then employed to show where the ANOVA was significant. An alpha level of 0.05 was used for all statistical tests. All analyses were corrected for multiple comparisons using the Bonferroni correction. Effect sizes for significant results are reported using *d*.

## 3. Results

### 3.1. Pilot Study Results

There were no significant correlations between pre-exercise breakfast size and baseline scores, post-exercise cognitive task outcomes or the appetite VAS or workload score. No associations were found between pre or post-exercise EI and BMI. Neither pre-, nor post-, exercise EI correlated significantly with TFEQ scores. Average waking time, exercise start time and exercise duration were 0755 h (range: 0615–0945 h), 0900 h (range: 0645–1015 h) and 62 min (25–135 min) respectively. The average pre-exercise breakfast size was 234 kcal (range: 0–743 kcal).

Energy intake at breakfast prior to exercise were significantly related to post-exercise mental fatigue (*r* (43) = −0.307; *p* = 0.043) and thirst (*r* (43) = −0.333; *p* = 0.027). As EI (kcal) increased, mental fatigue (Figure S1) and thirst ratings decreased. Energy intake at breakfast prior to exercise was also significantly related to post-exercise alertness (*r* (43) = 0.315; *p* = 0.037) and overall mood (*r* (43) = 0.372; *p* = 0.013). As EI (kcal) increased, alertness and overall mood increased (Figure S1). Energy intake (kcal) consumed at breakfast prior to exercise was significantly related to post-exercise EI (*r* (43) = 0.421; *p* =0.004) with a higher EI (kcal) consumed at breakfast, was associated with a higher post-exercise EI (Figure S2).

### 3.2. Main Study Results

#### 3.2.1. Cognitive Function

No significant results were found for laboratory-based computer cognitive task performance between the different treatment conditions up to 2 h post-exercise (Table S1).

**Figure 2:**
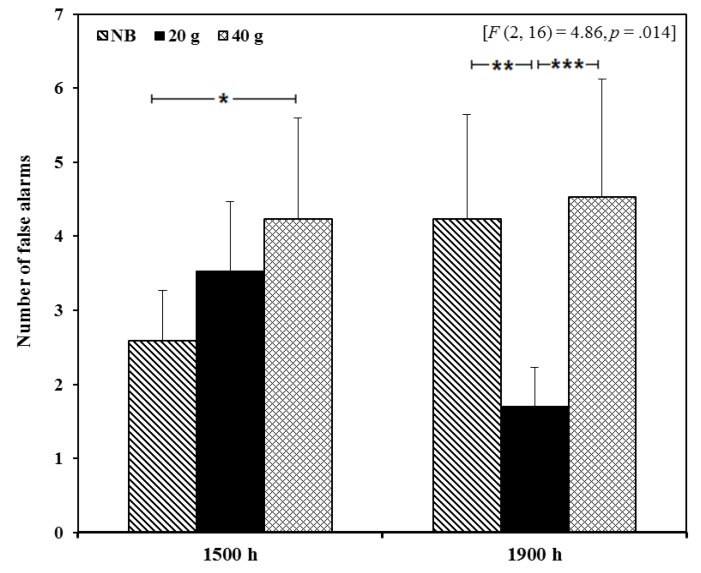
The effects of consuming breakfast cereal (20 g or 40 g) or no breakfast (NB) prior to exercise on RVIP false alarms 5 h (1500 h) and 9 h (1900 h) post-exercise, in habitually active females (*n* = 17). (* *p* < 0.05, ** *p* < 0.01, *** *p* < 0.001).

A significant breakfast × repetition interaction was observed for RVIP false alarms (errors) [*F* (2, 16) = 4.86, *p* = 0.014] when completed on the mobile phone. Comparisons revealed that at 1500 h participants made significantly less false alarms in the no breakfast (NB) condition compared to the 40 g breakfast condition (*p* = 0.019, *d* = 0.37). However, at 1900 h there were significantly less false alarm responses in the 20 g breakfast condition compared to the NB (*p* = 0.001, *d* = 0.58) and 40 g breakfast (*p* < 0.0001, *d* = 0.58) conditions (Figure 2, Table S1).

#### 3.2.2. Mood and Physical State

**Figure 3:**
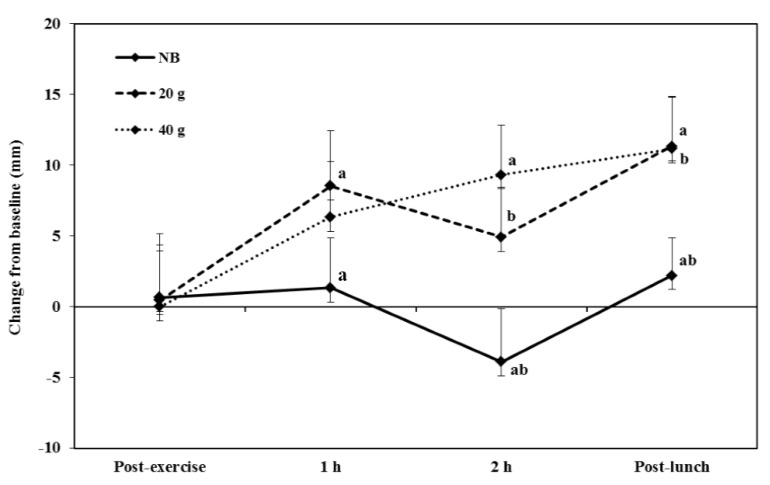
The effects of consuming breakfast cereal (20 g or 40 g) or no breakfast (NB) prior to exercise on feelings of relaxation immediately, 1 and 2 h post-exercise and post-lunch, in habitually active females (*n* = 24). Points which share a letter are significantly different from one another (* *p* < 0.05)

A significant breakfast x repetition interaction was observed for relaxed scores [*F* (2, 23) = 2.51, *p* = 0.025]. Comparisons revealed that at 1 h and 2 h post-exercise and post-lunch participants were significantly less relaxed in the NB condition compared to the 20 g breakfast condition (*p* = 0.013, *d* = 0.40, *p* = 0.003, *d* = 0.50 and *p* < 0.002, *d* = 0.60 respectively) and 40 g breakfast condition (2 h post-exercise and post-lunch only, *p* < 0.001, *d* = 0.75 and *p* = 0.002, *d* = 0.57 respectively; Figure 3, Table S2). No other significant differences were observed between conditions for the computer Mood and Physical State Visual Analogue Scales (see Table S2). There were also no significant differences observed between the NB, 40 g and 20 g breakfast conditions for RPE during exercise (10.8 ± 0.3, 10.7 ± 0.3 and 10.6 ± 0.3 respectively) or exercise enjoyment (53.8 ± 2.3, 54.6 ± 3.1, 58.7 ± 2.1 respectively).

**Figure 4:**
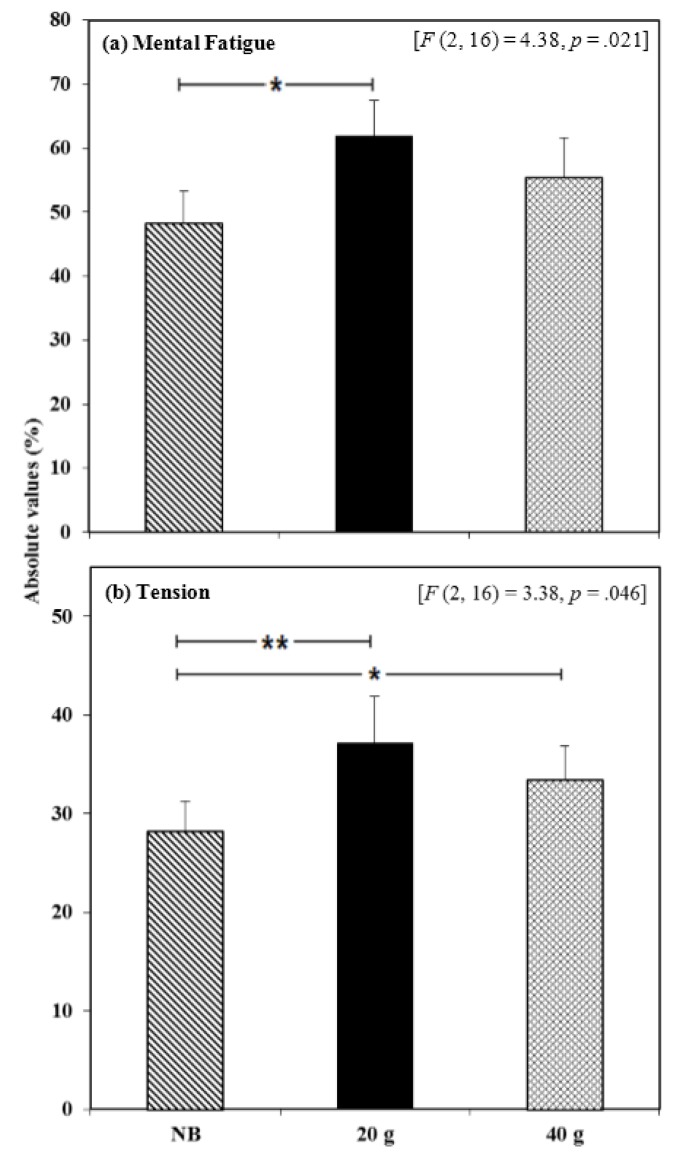
The effects of consuming breakfast cereal (20 g or 40 g) or no breakfast (NB) prior to exercise on ratings of (**a**) mental fatigue 5–9 h post-exercise and (**b**) tension 9 h post-exercise, in habitually active females (*n* = 17) (* *p* < 0.05, ** *p* < 0.001).

Significant effects were found for mental fatigue and tension ratings when completed using the mobile phone. A significant main effect of breakfast condition was observed for mental fatigue ratings when completed 5–9 h post-exercise [*F* (2, 16) = 4.38, *p* = 0.021]. Comparisons revealed that participants were significantly less mentally fatigued in the NB condition compared to the 20 g breakfast condition (*p* = 0.018, *d* = 0.62; Figure 4a, Table S2). A significant breakfast × repetition interaction was also observed for tension scores [*F* (2, 16) = 3.38, *p* = 0.046]. Comparisons revealed that at 1900 h participants were significantly less tense in the NB condition compared to the 20 g (*p* = 0.001, *d* = 0.55) and 40 g (*p* = 0.045, *d* = 0.38) breakfast conditions (Figure 4b, Table S2).

#### 3.2.3. Appetite

**Figure 5:**
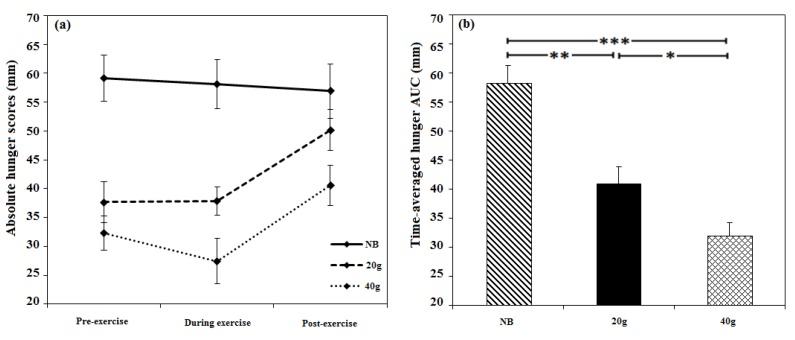
The effects of consuming breakfast cereal (20 g or 40 g) or no breakfast (NB) prior to exercise on pre-, during and post-exercise hunger in habitually active females (*n* = 24). Values are (**a**) absolute and (**b**) time-averaged AUC (* *p* < 0.05, ** *p* < 0.01, *** *p* <0.001).

Significant main effects of breakfast were observed for pre-during-post exercise hunger [*F* (2, 23) = 23.88, *p* < 0.0001], desire to eat [*F* (2, 23) = 23.11, *p* < 0.0001], fullness [*F* (2, 23) = 43.09, *p* < 0.0001] and satisfaction [*F* (2, 23) = 39.91, *p* < 0.0001] AUC ratings. Participant’s reported feeling significantly less hungry (*p* = 0.025, *d* = 0.68; Figure 5a) and felt they could eat less (*p* = 0.020, *d* = 0.59) after consuming the 40 g compared to 20 g breakfast (see Table S1). When they had consumed no breakfast compared to the 20 g and 40 g breakfasts participants reported feeling more hungry (*p* < 0.0001, *d* = 1.11 and *p* < 0.0001, d = 1.86 respectively; Figure 5b) and desired to eat more (*p* < 0.0001, *d* = 1.12 and *p* < 0.0001, *d* = 1.60 respectively) and felt less full (*p* < 0.0001, *d* = 1.63 and *p* < 0.0001, *d* = 2.09 respectively) and less satisfied (*p* < 0.0001, *d* = 1.67 and *p* < 0.0001, *d* = 1.79 respectively).

**Figure 6:**
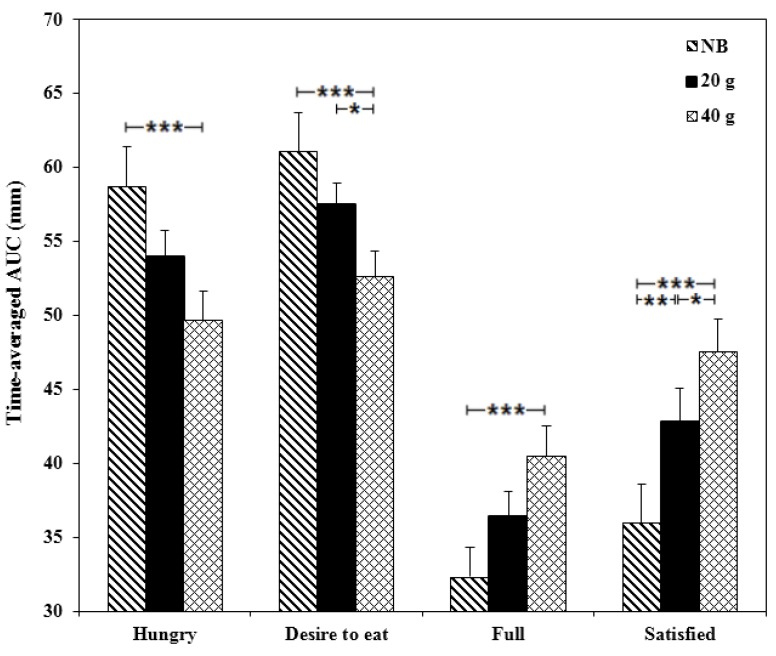
The effects of consuming breakfast cereal (20 g or 40 g) or no breakfast (NB) prior to exercise on hunger, desire to eat, fullness and satisfaction over a 3 h post-exercise period, in habitually active females (*n* = 24). Values are time-averaged AUC (* *p* < 0.05, ** *p* < 0.01, *** *p* < 0.001).

Significant main effects of breakfast were observed for post-exercise hunger [*F* (2, 23) = 6.60, *p* = 0.003], desire to eat [*F* (2, 23) = 6.49, *p* = 0.003], fullness [*F* (2, 23) = 6.20, *p* = 0.004] and satisfaction [*F* (2, 23) = 16.41, *p* < 0.0001; Figure 6]. When no breakfast was consumed compared to 40 g breakfast, participants reported feeling more hungry (*p* = 0.001, *d* = 0.78), desired to eat more (*p* = 0.001, *d* = 0.79) and felt less full (*p* = 0.001, *d* = 0.83 respectively). They were also less satisfied when no breakfast was consumed compared to both the 20 g and 40 g breakfasts (*p* = 0.001, *d* = 0.58 and *p* < 0.0001, *d* = 0.97 respectively). They also reported feeling that they felt they could eat significantly less (*p* = 0.044, *d* = 0.64) and were significantly more satisfied (*p* = 0.025, *d* = 0.43; Figure 6) after consuming the 40 g compared to 20 g breakfast (Table S3). There were no significant differences observed between conditions for subjective appetite between 1500 and 1900 h measured using the mobile phone (Table S3).

#### 3.2.4. Energy Intake

There was no significant difference between the conditions for EI during the ad libitum lunch or post-lunch (Table 2).

#### 3.2.5. Meal Liking

A significant main effect of breakfast was observed for the “breakfast liking” VAS [*F* (2, 23) = 106.83, *p* < 0.0001]. However, whereas participants “liked” consuming the 20 g and 40 g breakfast significantly more than consuming no breakfast (*p* < 0.0001, *d* = 3.62 and *p* < 0.0001, *d* = 3.20 respectively), both the 20 g and 40 g breakfast were liked equally as much (Table 2). There was no significant differences between conditions for the “lunch liking” VAS (Table 2).

**Table 2:** Rate of perceived exertion during exercise, exercise enjoyment, meal liking and energy intake (lunch, post-lunch and net) mean values (*n* = 24).

Measure	Condition	Mean Value		
Breakfast Liking (mm)	NB	19.4 *^, **†**^	±	2.7
	20 g	68.9	±	3.0
	40 g	67.9	±	3.5
Lunch Liking (mm)	NB	74.5	±	3.0
	20 g	76.5	±	2.2
	40 g	75.8	±	2.4
Lunch Energy Intake (kcal)	NB	763.2	±	37.2
	20 g	786.2	±	37.1
	40 g	778.7	±	38.8
Post-lunch Energy Intake (kcal; *n* = 22)	NB	1000.8	±	76.9
	20 g	1055.9	±	105.6
	40 g	1067.5	±	114.5
Net Energy Intake (kcal; *n* = 22)	NB	1780.14	±	80.59
	20 g	1957.21	±	114.64
	40 g	2088.57	±	132.30

Means ± SEM are presented; * Mean value was significantly different from 40 g breakfast (*p* < 0.05); **^†^** Mean value was significantly different from 20 g breakfast (*p* < 0.05).

## 4. Discussion

### 4.1. Pilot Study

A preliminary, single-day field study was conducted to assess whether energy intake at breakfast prior to morning exercise influenced cognitive function, mood, appetite and EI for the remainder of the day. An increase in EI at breakfast was associated with lower mental fatigue, higher alertness and EI and better overall mood post-exercise. The limitations of conducting a field study are obvious; a lack of control over the environment in which the study is completed which may lead to confounding results and the inability to detect subtle effects which may only become apparent in a more controlled environment. Participants notoriously under-report when asked to complete a food diary, decreasing the internal validity of the data [38]. For this reason, collecting additional data on a subsequent day would have been preferable. However, these results did suggest that breakfast size before exercise may influence energy intake and mood in active females, warranting further investigation in a randomised controlled trial as subsequently discussed.

### 4.2. Main Study

Beneficial pre-exercise nutritional practices for females exercising for weight control or mood and cognitive benefits have not been well established. The main study presented in this paper aimed to assess the effect of breakfast size prior to exercise on post-exercise cognitive performance, mood and appetite in healthy, habitually active females. Breakfast consumption compared to breakfast omission prior to exercise improved subjective hunger ratings, irrespective of size. Breakfast was also favourable for some mood benefits in the hours following exercise, but consuming a smaller breakfast (118 kcals) prevented mid-afternoon cognitive decrements associated with consuming a larger breakfast (236 kcals).

Feelings of relaxation were enhanced in the initial post-exercise period when either breakfast was consumed, a positive finding given that women sight stress reduction as a main reason for exercise [9]. Consuming breakfast replicated the usual routine of this sample and some evidence suggests that habitual breakfast habits may influence mood responses to acute breakfast consumption [39,40], which may explain this finding. In addition, past research suggests that CHO intake may reduce the effects of energy depletion in the brain by attenuating the synthesis of certain metabolites and neurotransmitters [41]. Previously, a higher concentration of plasma glucose was observed immediately post-exercise when CHO, compared to placebo, had been consumed during a moderate intensity 2 h cycle, which corresponded with increased feelings of pleasure in males [42]. Foster *et al.*, (2007) also found higher blood glucose accompanied increases in alertness and contentment when breakfast was consumed rather than omitted [43]; consuming a meal containing CHO prior to exercise may contribute to post-exercise mood state by increasing pre, and therefore post, exercise glycogen stores [17]. 

Worse performance on the RVIP task was observed mid-afternoon when the larger breakfast was consumed compared to the smaller breakfast or no breakfast and in the evening fewer RVIP errors were seen following consumption of the smaller breakfast compared to the other conditions. Although previous data suggests working memory is improved by prior breakfast consumption (for review see [27]), our data suggests that when consumed prior to exercise, a smaller, rather than larger, breakfast avoids detriments in working memory later in the day. These results somewhat mirror those found by Nabb and Benton (1996), who reported that memory score negatively correlated with the caloric content of the breakfast administered, attributing this to only minor increases in blood glucose following a smaller meal [44]. Indeed, recently Zilberter and Zilberter (2013) suggested that the belief that consuming breakfast is beneficial for cognitive function may be incorrect [45]. They argued that consumption of a low-CHO, high-fat breakfast or indeed omitting breakfast produces the most stable metabolic response, a factor which appears to contribute positively to cognitive function possibly via a neuroprotective effect. However, even though fasting may lead to a more stable metabolic response acutely, there is evidence that insulin sensitivity and glucose response to a meal are improved when breakfast has been consumed beforehand both acutely [46] and regularly [8].

It is perhaps surprising that cognitive differences were observed between the conditions when tasks were completed on the mobile phone but not when completed on a computer in the laboratory. Whilst the lack of cognitive effects in the initial post-exercise period does mirror previous laboratory based data [15,16] few studies have looked at the effect of breakfast consumption on cognitive function in the evening. A delayed effect of breakfast on cognitive performance, or of course a second-meal effect, whereby the effects of breakfast on cognitive performance only become apparent after the next meal has been consumed, are plausible given that the glycaemic index of an evening meal has been shown to affect cognitive function post-breakfast breakfast the following morning [34]. It has also been suggested that if breakfast is consumed regularly, omitting breakfast occasionally may not have a negative impact on cognitive performance; rather these effects may only be seen if breakfast is omitted over a long period of time [47]. In the current study, consuming breakfast lowered subjective appetite at every time point following breakfast up until immediately after lunch with the greatest differences observed between the NB and 40 g breakfast conditions, as expected. Whilst these results are not considered novel when viewed in isolation, the study also aimed to draw parallels between subjective appetite sensations and mood state and cognitive function. Indeed, the superior appetite profile seen following breakfast concurred with an improvement in at least one facet of mood at most time points throughout the study day; we reported a similar effect in a previous study in male subjects when enhanced appetite control occurred concurrently with lower mental fatigue ratings [16]. Breakfast can also positively influence appetite responses to a second feeding [46] but our data showed no effect of breakfast on appetite beyond immediately post-lunch; any effects were likely masked due to the ad libitum nature of the lunch meal.

Research shows that breakfast consumption can also reduce subsequent EI [46,48] although the effect of exercise on this parameter is debatable [49,50]. Previous data from our laboratory [12] revealed that breakfast consumption (451 kcals) compared to omission before exercise improved subjective appetite control, but did not affect EI at lunch following exercise in males. Similarly, lower subjective appetite ratings did not coincide with a reduction in EI at lunch or post-lunch in the current study. This could be due to a lack of power to detect EI effects when administering an ad libitum meal although other explanations should be considered. The majority of the sample in the current study were restrained eaters. The high prevalence of eating restraint in females [51] is consistently highlighted as a problem in this area of research, although there is data suggesting dietary restraint is not a reliable predictor of EI [52,53]. It should also be considered that post-lunch, participants were able to resume their normal diet; there may of course be an interactive effect of breakfast and exercise on subsequent EI, but not one which is robust enough to override everyday habitual behaviours [54]. It is also important to acknowledge here the well-known complications in gathering accurate food-intake information using food diaries [38,55], and this data should be viewed with this in consideration.

A limitation this study was the unconstrained environment in which the mobile phone tasks were completed and a variety of nutritional, behavioural and emotional factors may have influenced task performance and mood state at these times; this should be considered when interpreting the apparent negative effect that the smaller breakfast had on tension and mental fatigue in the evening. Nevertheless, this novel method is considered suitable for data collection of this nature [56] and the “free-living” data it provides is lacking in the literature and is useful to guide the direction of research and formulate future hypotheses. The breakfasts administered provided approximately 5% and 10% of the energy needs of a typical adult female, lower than recommended (between 20% and 35% of total daily energy needs [57]). However, the larger of the breakfasts administered in the current study was based on data from the pilot study where the average breakfast consumed was just 234 kcal and therefore perhaps reflects that typically consumed by the population of interest. It should also be noted that the both studies described in this paper used predominately student samples; therefore, this data may not be as valid for other populations.

It should certainly be considered that the importance of eating a substantial breakfast regularly for other health benefits has been established and the results from this study which show some benefits of a smaller breakfast may not be applicable to the majority. However, if post-exercise mood and appetite benefits can be attained by eating a small, quick breakfast, which is equally liked compared to consuming a larger breakfast, then those who skip breakfast prior to exercise due to lack of time or to avoid discomfort during exercise may be able to incorporate a breakfast such as this into their morning exercise routine successfully. Testing of this theory in a controlled trial using this particular population would be a logical next step in this area of research. Additional research is needed to explore further how pre-exercise nutrition may impact on post-exercise wellbeing and performance of daily activities. This research could focus on the glycaemic index (GI) or macronutrient content of the breakfast consumed; initial data suggests that the GI of breakfast consumed prior to exercise may affect cognition depending on the domain being observed [58] and that a high-energy CHO drink does not appear to elicit positive mood changes during exercise in women [59]. It would also be interesting to investigate whether a breakfast split between pre and post-exercise would elicit further benefits.

## 5. Conclusions

In summary, we believe this to be the first controlled intervention study to demonstrate that breakfast consumption prior to exercise can have a positive, although transient, influence on some aspects of mood and result in superior appetite control after exercise in an active female sample. A pre-exercise breakfast of approximately 118 kcal can improve mood and appetite control post-exercise, although further research is needed before this can be considered a recommendation.

## Supplementary Files

Supplementary File 1
