# Supplementary material for: The Effect of Breakfast Prior to Morning Exercise on Cognitive Performance, Mood and Appetite Later in the Day in Habitually Active Women
Source: Nutrients. 2015 Jul 14;7(7):5712–32. doi: 10.3390/nu7075250 (PMC4517027; doi:10.3390/nu7075250)
Supplement: Supplementary File 1 [file nutrients-07-05250-s001.docx]

**Supplementary Material**


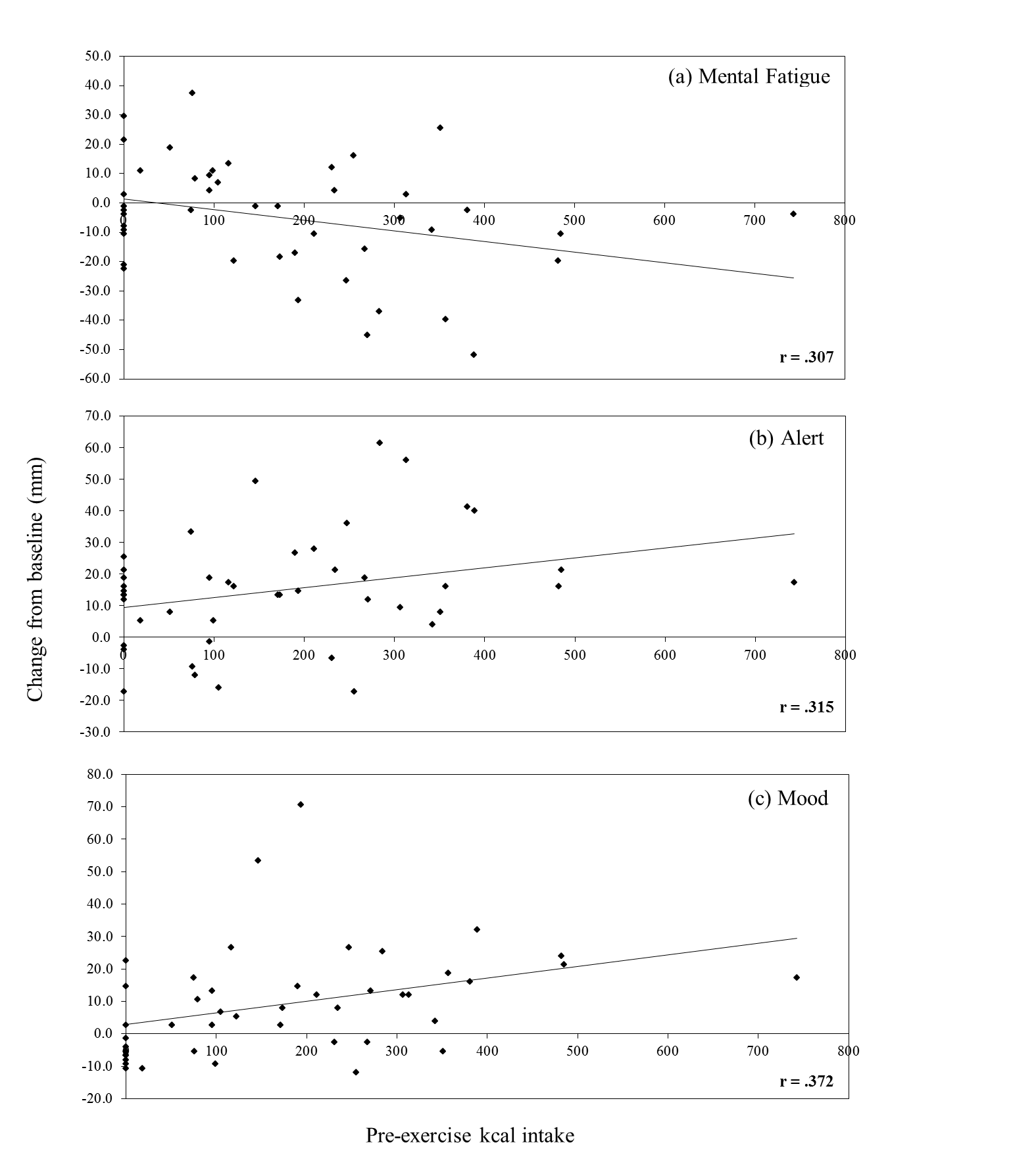


**Figure S1.** The effects of breakfast size (kcal) prior to morning exercise on (**a**) Mental Fatigue; (**b**) Alertness; and (**c**) Mood, in habitually active females (*n* = 45). X-axis values are change from baseline.


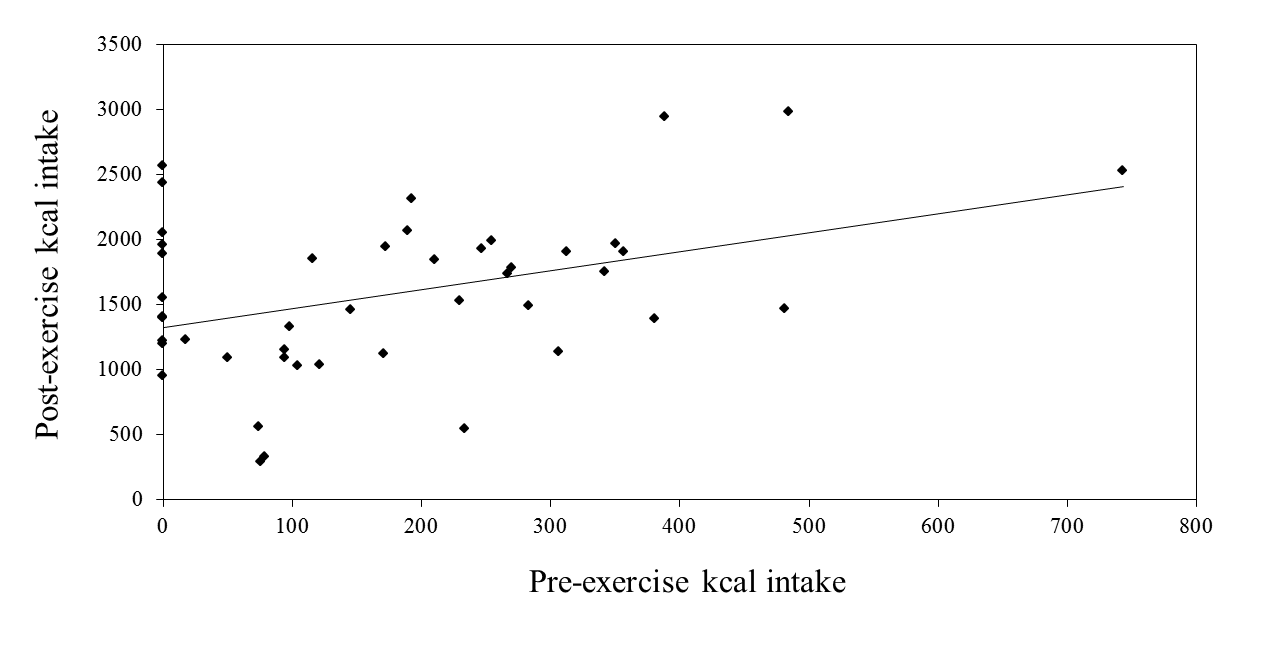


**Figure S2.** The effects of breakfast size (kcal) prior to morning exercise on energy intake post-exercise, in habitually active females (*n* = 42).

**Table S1.** Baseline and change from baseline scores for each cognitive measure for each treatment condition.

| **Measure** | **Condition** | **COMPASS Tasks (Change from Baseline)** | | | | | | **Mobile Phone Tasks (Absolute Values)** | | | | | |
| --- | --- | --- | --- | --- | --- | --- | --- | --- | --- | --- | --- | --- | --- |
|  |  | **Baseline Score** | **Pre-Exercise** | **Post-Exercise** | **1 h Post-Exercise** | **2 h Post-Exercise** | **Post-Lunch** | **1500 h** | | | **1900 h** | | |
| Four Choice Reaction Time (ms) | NB | 457.3 ± 13.2 | 25.7 ± 11.4 | 18.4 ± 11.9 | 29.9 ± 12.9 | 32.7 ± 13.4 | 50.5 ± 18.1 | 426.8 ± 15.0 | | | 428.4 ± 14.2 | | |
|  | 20 g | 471.6 ± 15.7 | 3.1 ± 10.1 | −10.4 ± 9.7 | 3.1 ± 10.2 | 2.6 ± 10.6 | 13.1 ± 13.7 | 431.3 ± 14.2 | | | 436.5 ± 17.3 | | |
|  | 40 g | 459.2 ± 12.1 | 24.4 ± 13.3 | −5.9 ± 11.3 | 4.6 ± 10.7 | 9.7 ± 10.8 | 19.6 ± 7.9 | 423.1 ± 17.8 | | | 437.9 ± 24.5 | | |
| Four Choice Reaction Time Correct RT (ms) | NB | 458.21 ± 13.22 | 24.7 ± 11.3 | 17.7 ± 11.9 | 29.6 ± 12.8 | 32.7 ± 13.5 | 49.5 ± 18.1 | 385.4 ± 11.7 | | | 375.5 ± 10.4 | | |
|  | 20 g | 471.53 ± 15.68 | 3.3 ± 10.1 | −10.3 ± 9.8 | 2.9 ± 10.3 | 3.3 ± 10.8 | 13.2 ± 13.8 | 392.3 ± 12.9 | | | 395.3 ± 12.4 | | |
|  | 40 g | 459.3 ± 12.09 | 25.1 ± 13.2 | −5.8 ± 11.2 | 5.3 ± 10.6 | 9.9 ± 10.8 | 20.0 ± 7.8 | 390.8 ± 15.5 | | | 397.9 ± 21.9 | | |
| Four Choice Reaction Time Accuracy (%) | NB | 98.8 ± 0.4 | 0.5 ± 0.4 | 0.8 ± 0.3 | 0.4 ± 0.3 | 0.3 ± 0.6 | 0.5 ± 0.4 | 385.4 ± 11.7 | | | 375.5 ± 10.4 | | |
|  | 20 g | 99.6 ± 0.2 | 0.1 ± 0.3 | 0.1 ± 0.3 | −0.5 ± 0.4 | −0.5 ± 0.4 | −0.3 ± 0.5 | 392.3 ± 12.9 | | | 395.3 ± 12.4 | | |
|  | 40 g | 99.9 ± 0.1 | −1.0 ± 0.4 | −0.7 ± 0.3 | −0.8 ± 0.4 | −0.5 ± 0.4 | −0.4 ± 0.3 | 390.8 ± 15.5 | | | 397.9 ± 21.9 | | |
| Stroop accuracy (%) | NB | 97.8 ± 0.3 | -0.3 ± 0.4 | 0.0 ± 0.3 | −1.1 ± 0.4 | −1.3 ± 0.5 | −0.9 ± 0.5 |  |  |  |  |  |  |
|  | 20 g | 97.0 ± 0.5 | 0.2 ± 0.4 | −0.3 ± 0.6 | −0.3 ± 0.4 | 0.3 ± 0.5 | −0.1 ± 0.4 |  |  |  |  |  |  |
|  | 40 g | 97.5 ± 0.3 | 0.0 ± 0.4 | −0.2 ± 0.4 | −0.8 ± 0.3 | −0.5 ± 0.4 | −0.3 ± 0.4 |  |  |  |  |  |  |
| Stroop Overall RT (ms) | NB | 621.9 ± 11.5 | −0.3 ± 8.3 | −18.7 ± 6.4 | −1.0 ± 8.8 | −17.0 ± 9.2 | −5.9 ± 12.6 |  |  |  |  |  |  |
|  | 20 g | 631.4 ± 17.1 | −25.3 * ± 10.7 | −29.9 ± 8.2 | −22.2 ± 10.8 | −29.0 ± 11.6 | −20.7 ± 9.8 |  |  |  |  |  |  |
|  | 40 g | 620.6 ± 14.0 | 0.3 ± 7.6 | −14.0 ± 8.8 | −12.1 ± 6.8 | −16.3 ± 9.4 | −6.5 ± 10.8 |  |  |  |  |  |  |
| Stroop Correct RT (ms) | NB | 622.82 ± 11.66 | −1.5 ± 7.8 | −18.6 ± 6.6 | −2.0 ± 8.3 | −16.6 ± 9.3 | −6.5 ± 12.6 |  |  |  |  |  |  |
|  | 20 g | 631.63 ± 17.18 | −24.7 * ± 11.0 | −29.9 ± 8.3 | −21.9 ± 10.8 | −28.1 ± 11.8 | −19.7 ± 9.7 |  |  |  |  |  |  |
|  | 40 g | 620.49 ± 14.16 | 1.1 ± 7.8 | −13.5 ± 8.6 | −12.1 ± 7.1 | −15.2 ± 9.6 | −5.3 ± 10.7 |  |  |  |  |  |  |
| Stroop Congruent Correct (%) | NB | 97.782 ± 0.653 | 0.5 ± 0.7 | −0.3 ± 0.8 | −0.3 ± 0.8 | −1.1 ± 0.8 | 0.2 ± 0.7 |  |  |  |  |  |  |
|  | 20 g | 97.41 ± 0.612 | 0.1 ± 0.8 | −0.5 ± 0.8 | −0.1 ± 0.5 | 0.1 ± 0.6 | 0.1 ± 0.6 |  |  |  |  |  |  |
|  | 40 g | 97.452 ± 0.542 | −0.4 ± 0.7 | −0.5 ± 0.7 | −0.5 ± 0.7 | −0.1 ± 0.8 | −0.4 ± 0.8 |  |  |  |  |  |  |
| Stroop Incongruent correct (%) | NB | 97.735 ± 0.37 | −0.7 ± 0.7 | 0.1 ± 0.4 | −1.4 ± 0.6 | −1.3 ± 0.6 | −1.5 ± 0.7 |  |  |  |  |  |  |
|  | 20 g | 96.766 ± 0.6 | 0.4 ± 0.5 | −0.2 ± 0.8 | −0.3 ± 0.7 | 0.4 ± 0.6 | −0.2 ± 0.5 |  |  |  |  |  |  |
|  | 40 g | 97.467 ± 0.427 | 0.3 ± 0.6 | 0.0 ± 0.7 | −0.9 ± 0.5 | −0.6 ± 0.7 | −0.2 ± 0.6 |  |  |  |  |  |  |
| Stroop Congruent RT (ms) | NB | 590.49 ± 11.25 | 18.1 **†** ± 10.3 | −13.5 ± 8.4 | 10.2 ± 11.3 | 2.3 ± 12.9 | −5.6 ± 12.7 |  |  |  |  |  |  |
|  | 20 g | 609.28 ± 16.37 | −19.9 ± 13.2 | −33.3 ± 10.1 | −24.2 ± 9.5 | −22.4 ± 12.6 | −26.1 ± 12.5 |  |  |  |  |  |  |
|  | 40 g | 591.97 ± 10.3 | 7.8 ± 7.9 | −2.5 ± 12.3 | −7.0 ± 7.1 | −1.9 ± 8.0 | −5.3 ± 12.7 |  |  |  |  |  |  |

**Table S1.** *Cont.*

| **Measure** | **Condition** | **COMPASS Tasks (Change from Baseline)** | | | | | | **Mobile Phone Tasks (Absolute Values)** | | | | | |
| --- | --- | --- | --- | --- | --- | --- | --- | --- | --- | --- | --- | --- | --- |
|  |  | **Baseline Score** | **Pre-Exercise** | **Post-Exercise** | **1 h Post-Exercise** | **2 h Post-Exercise** | **Post-Lunch** | **1500 h** | | | **1900 h** | | |
| Stroop Incongruent RT (ms) | NB | 636.91 ± 12.99 | −10.1 ± 9.8 | −21.6 ± 7.5 | −6.1 ± 10.1 | −25.2 ± 8.3 | −5.7 ± 13.5 |  |  |  |  |  |  |
|  | 20 g | 643.01 ± 18.07 | −28.5 ± 11.2 | −28.2 ± 9.1 | −21.8 ± 12.4 | −32.6 ± 12.2 | −19.4 ± 10.2 |  |  |  |  |  |  |
|  | 40 g | 634.34 ± 16.16 | −3.0 ± 9.3 | −17.5 ± 9.1 | −15.6 ± 8.3 | −23.6 ± 11.2 | −7.2 ± 11.2 |  |  |  |  |  |  |
| Stroop Correct Congrue RT (ms) | NB | 591.24 ± 11.35 | 18.3 **†** ± 10.5 | −13.4 ± 8.7 | 10.2 ± 10.8 | 3.8 ± 13.2 | −6.0 ± 12.4 |  |  |  |  |  |  |
|  | 20 g | 610.04 ± 16.74 | −20.0 ± 14.1 | −32.6 ± 10.7 | −24.7 ± 9.9 | −21.1 ± 12.9 | −24.9 ± 13.1 |  |  |  |  |  |  |
|  | 40 g | 592.88 ± 10.4 | 7.8 ± 8.2 | −2.8 ± 12.4 | −6.7 ± 7.5 | −1.4 ± 8.1 | −4.8 ± 13.0 |  |  |  |  |  |  |
| Stroop Correct Incongruent RT (ms) | NB | 637.72 ± 13.23 | −11.7 ± 9.2 | −21.4 ± 7.6 | −7.3 ± 9.7 | −25.1 ± 8.3 | −5.6 ± 13.9 |  |  |  |  |  |  |
|  | 20 g | 643.11 ± 18.12 | −27.8 ± 11.3 | −28.7 ± 9.2 | −21.0 ± 12.3 | −32.0 ± 12.3 | −18.3 ± 10.0 |  |  |  |  |  |  |
|  | 40 g | 633.65 ± 16.39 | −1.8 ± 9.5 | −16.5 ± 9.1 | −15.5 ± 8.8 | −22.1 ± 11.6 | −5.8 ± 11.2 |  |  |  |  |  |  |
| NBack accuracy (%) | NB | 91.1 ± 1.6 | −1.7 **†** ± 1.2 | −0.9 ± 1.5 | −1.0 ± 1.5 | 1.3 ± 1.2 | −2.8 ± 1.4 | 89.7 * ± 1.1 | | | 88.9 ± 1.6 | | |
|  | 20 g | 87.7 ± 1.8 | 3.23 ± 1.5 | 1.4 ± 1.6 | 1.3 ± 1.7 | 2.8 ± 1.7 | 2.1 ± 2.0 | 89.6 * ± 1.4 | | | 87.3 ± 1.5 | | |
|  | 40 g | 88.3 ± 1.8 | 0.5 ± 2.1 | 0.9 ± 1.9 | 2.4 ± 1.8 | 2.3 ± 1.6 | 3.4 ± 1.8 | 87.7 ± 1.6 | | | 89.1 ± 1.3 | | |
| NBack RT (ms) | NB | 741.6 ± 38.5 | −10.7 ± 19.4 | −60.5 ± 20.4 | −61.3 ± 21.8 | −77.6 ± 24.1 | −87.2 ± 28.6 | 831.3 ± 62.3 | | | 792.0 ± 56.2 | | |
|  | 20 g | 724.2 ± 36.5 | −20.0 ± 27.0 | −54.1 ± 18.2 | −41.3 ± 21.2 | −82.9 ± 32.6 | −71.6 ± 24.4 | 844.2 ± 59.4 | | | 778.0 ± 56.7 | | |
|  | 40 g | 724.6 ± 35.8 | 24.1 ± 28.3 | −69.5 ± 19.5 | −70.2 ± 21.1 | −75.6 ± 21.2 | −78.1 ± 26.3 | 832.5 ± 82.6 | | | 800.6 ± 76.0 | | |
| NBack Missed Sequences (number) | NB | 0.2 ± 0.1 | 0.2 **†** ± 0.2 | 0.2 ± 0.2 | 0.2 ± 0.1 | 0.2 ± 0.1 | 0.1 ± 0.1 |  |  |  |  |  |  |
|  | 20 g | 0.8 ± 0.4 | −0.6 ± 0.4 | −0.6 ± 0.4 | −0.3 ± 0.4 | −0.8 ± 0.4 | −0.7 ± 0.4 |  |  |  |  |  |  |
|  | 40 g | 0.4 ± 0.2 | 0.1 ± 0.2 | 0.0 ± 0.2 | −0.2 ± 0.2 | 0.0 ± 0.2 | −0.2 ± 0.1 |  |  |  |  |  |  |
| NBack Correct RT (ms) | NB | 730.9 ± 38.8 | −6.7 ± 20.3 | −63.6 ± 22.6 | −53.0 ± 23.2 | −60.8 ± 26.3 | −83.8 ± 30.2 | 17.3 ± 1.1 | | | 16.6 ± 0.9 | | |
|  | 20 g | 730.3 ± 37.5 | −33.1 ± 23.7 | −64.8 ± 20.1 | −47.9 ± 19.9 | −91.9 ± 31.5 | −79.1 ± 25.6 | 16.2 ± 1.1 | | | 14.1 ± 1.2 | | |
|  | 40 g | 722.0 ± 35.8 | 25.9 ± 30.0 | −68.5 ± 18.9 | −65.2 ± 18.5 | −77.6 ± 21.4 | −74.5 ± 23.3 | 16.2 ± 1.1 | | | 15.5 ± 1.2 | | |
| NBack Correct Target (%) | NB | 82.7 ± 3.5 | −2.6 ± 2.9 | −3.0 ± 2.9 | −3.0 ± 3.6 | 2.3 ± 2.9 | −7.5 ± 4.1 | 5.6 ± 0.9 | | | 6.5 ± 1.1 | | |
|  | 20 g | 77.9 ± 3.6 | 1.7 ± 3.5 | −0.5 ± 3.4 | 0.8 ± 3.4 | 2.6 ± 3.2 | −0.7 ± 3.4 | 5.9 ± 1.0 | | | 8.0 ± 1.3 | | |
|  | 40 g | 76.8 ± 3.8 | 1.9 ± 3.8 | 3.5 ± 3.7 | 3.8 ± 4.1 | 6.2 ± 3.8 | 6.5 ± 4.1 | 6.3 ± 1.0 | | | 6.5 ± 1.2 | | |
| NBack Target RT (ms) | NB | 791.1 ± 47.4 | −0.7 ± 0.9 | 0.9 ± 1.0 | 0.5 ± 1.3 | 1.6 ± 1.0 | 0.2 ± 0.8 | 767.8 ± 55.1 | | | 741.6 ± 45.4 | | |
|  | 20 g | 750.9 ± 51.8 | 1.8 ± 1.1 | 0.1 ± 1.2 | 0.5 ± 1.1 | −0.1 ± 1.3 | 0.9 ± 1.3 | 816.5 ± 57.4 | | | 779.9 ± 62.2 | | |
|  | 40 g | 747.7 ± 46.6 | 0.3 ± 1.4 | −0.1 ± 1.3 | 1.4 ± 1.1 | 0.4 ± 1.2 | 1.5 ± 1.1 | 796.1 ± 65.9 | | | 773.5 ± 71.9 | | |

**Table S1.** *Cont.*

| **Measure** | **Condition** | **COMPASS Tasks (Change from Baseline)** | | | | | | **Mobile Phone Tasks (Absolute Values)** | |
| --- | --- | --- | --- | --- | --- | --- | --- | --- | --- |
|  |  | **Baseline Score** | **Pre-Exercise** | **Post-Exercise** | **1 h Post-Exercise** | **2 h Post-Exercise** | **Post-Lunch** | **1500 h** | **1900 h** |
| NBack Correct Non-target (%) | NB | 96.0 ± 0.8 | −16.4 ± 31.8 | −73.5 ± 33.2 | −74.7 ± 26.4 | −108.6 ± 32.1 | −109.5 ± 41.0 | 76.9 * ± 0.9 | 76.7 ± 1.0 |
|  | 20 g | 95.6 ± 1.1 | −20.4 ± 38.2 | −33.1 ± 31.3 | −18.8 ± 44.7 | −102.0 ± 44.5 | −89.0 ± 42.1 | 77.8 * ± 0.8 | 77.6 ± 0.9 |
|  | 40 g | 95.4 ± 0.8 | 69.2 ± 36.4 | −62.8 ± 26.6 | −70.1 ± 29.1 | −48.9 ± 27.3 | −81.9 ± 36.5 | 75.7 ± 1.0 | 77.9 ± 0.6 |
| NBack  Non-target RT (ms) | NB | 725.3 ± 39.1 | −2.6 ± 24.2 | −47.2 ± 24.3 | −49.0 ± 24.5 | −57.3 ± 27.5 | −74.4 ± 26.4 | 894.8 ± 77.2 | 842.5 ± 73.2 |
|  | 20 g | 740.2 ± 37.4 | −41.2 ± 24.2 | −86.3 ± 21.5 | −66.0 ± 16.5 | −102.8 ± 28.5 | −88.5 ± 25.9 | 871.9 ± 81.6 | 776.1 ± 60.7 |
|  | 40 g | 728.2 ± 35.8 | 4.4 ± 30.2 | −74.2 ± 20.5 | −77.5 ± 22.9 | −91.5 ± 21.9 | −83.0 ± 25.3 | 868.9 ± 104.4 | 827.6 ± 85.9 |
| RVIP accuracy (%) | NB | 49.7 ± 4.6 | −2.1 ± 2.9 | 1.5 ± 2.5 | −2.8 ± 2.4 | −0.4 ± 2.6 | −2.6 ± 2.2 | 19.2 ± 2.5 | 19.4 ± 2.4 |
|  | 20 g | 52.7 ± 4.3 | −4.9 ± 2.1 | 0.6 ± 2.7 | −1.3 ± 2.7 | 1.5 ± 2.4 | −3.0 ± 2.5 | 19.9 ± 2.2 | 21.1 ± 2.3 |
|  | 40 g | 53.6 ± 5.1 | −1.5 ± 1.8 | 2.0 ± 2.4 | −2.6 ± 1.9 | 0.7 ± 1.9 | −5.3 ± 2.7 | 20.1 ± 2.4 | 19.4 ± 2.2 |
| RVIP reaction time (ms) | NB | 468.1 ± 23.0 | 24.5 ± 21.6 | 15.0 ± 21.2 | 14.8 ± 22.4 | 31.1 ± 22.2 | 12.5 ± 19.4 | 468.2 ± 14.7 | 460.6 ± 11.2 |
|  | 20 g | 493.1 ± 10.8 | 3.8 ± 8.0 | −8.7 ± 7.4 | 0.4 ± 10.8 | −3.2 ± 9.3 | −2.4 ± 9.0 | 495.4 ± 12.2 | 466.2 ± 11.0 |
|  | 40 g | 458.1 ± 22.1 | 12.4 ± 6.5 | 26.4 ± 34.1 | 3.9 ± 8.9 | −7.5 ± 6.9 | −2.2 ± 8.6 | 474.5 ± 9.6 | 474.9 ± 10.8 |
| RVIP false alarms (number) | NB | 0.5 ± 0.2 | −0.1 ± 0.2 | −0.3 ± 0.2 | −0.1 ± 0.2 | 0.3 ± 0.4 | 0.0 ± 0.2 | 2.6 * ± 0.7 | 4.2 **†** ± 1.4 |
|  | 20 g | 0.8 ± 0.2 | 0.0 ± 0.3 | −0.1 ± 0.2 | −0.1 ± 0.2 | −0.2 ± 0.2 | −0.3 ± 0.3 | 3.5 ± 0.9 | 1.7 * ± 0.5 |
|  | 40 g | 0.5 ± 0.2 | 0.2 ± 0.3 | −0.2 ± 0.2 | 0.0 ± 0.2 | −0.2 ± 0.3 | 0.2 ± 0.2 | 4.2 ± 1.4 | 4.5 ± 1.6 |

Means ± SEM are presented; * Mean value was significantly different from 40 g breakfast; **†** Mean value was significantly different from 20 g breakfast.

**Table S2.** Baseline and change from baseline or absolute scores for each mood measure for each treatment condition.

| **Measure** | **Condition** | **COMPASS Tasks (Change from Baseline)** | | | | | | **Mobile Phone Tasks (Absolute Values)** | |
| --- | --- | --- | --- | --- | --- | --- | --- | --- | --- |
|  |  | **Baseline Score** | **Pre-Exercise** | **Post-Exercise** | **1 h Post-Exercise** | **2 h Post-Exercise** | **Post-Lunch** | **1500 h** | **1900 h** |
| MPSVAS Relaxed | NB | 58.5 ± 3.5 | −3.5 **†** ± 3.6 | 0.7 ± 4.5 | 1.3 * ± 3.5 | −3.9 * ± 3.7 | 2.2 * ± 2.6 | 58.1 ± 4.3 | 55.5 ± 3.9 |
|  | 20 g | 54.2 ± 3.2 | 4.5 ± 3.8 | 0.5 ± 3.9 | 8.5 ± 3.9 | 4.9 ± 3.5 | 11.3 ± 3.5 | 58.4 ± 4.4 | 52.2 ± 5.3 |
|  | 40 g | 52.6 ± 3.8 | 8.4 ± 3.6 | 0.0 ± 3.9 | 6.3 ± 4.0 | 9.3 ± 3.5 | 11.2 ± 3.7 | 59.1 ± 4.9 | 56.5 ± 4.4 |
| Alert | NB | 41.6 ± 3.4 | 4.6 ± 3.7 | 17.0 ± 3.3 | 10.0 ± 4.4 | 2.9 ± 4.9 | 12.8 ± 4.8 | 50.8 ± 3.8 | 52.9 ± 4.1 |
|  | 20 g | 44.4 ± 3.1 | 7.9 ± 3.0 | 12.9 ± 4.3 | 6.4 ± 4.5 | 6.1 ± 5.3 | 13.8 ± 4.7 | 52.2 ± 4.0 | 50.1 ± 3.8 |
|  | 40 g | 44.8 ± 3.0 | 6.4 ± 3.9 | 13.0 ± 3.4 | 6.9 ± 3.6 | 10.9 ± 3.7 | 14.0 ± 3.9 | 54.8 ± 3.7 | 53.9 ± 2.9 |

**Table S2.** *Cont.*

| **Measure** | **Condition** | **COMPASS Tasks (Change from Baseline)** | | | | | | **Mobile Phone Tasks (Absolute Values)** | |
| --- | --- | --- | --- | --- | --- | --- | --- | --- | --- |
|  |  | **Baseline Score** | **Pre-Exercise** | **Post-Exercise** | **1 h Post-Exercise** | **2 h Post-Exercise** | **Post-Lunch** | **1500 h** | **1900 h** |
| Jittery | NB | 24.8 ± 3.3 | −0.6 ± 2.3 | 4.0 ± 3.5 | 2.4 ± 3.4 | 2.8 ± 2.3 | −0.9 ± 2.1 | 26.4 ± 3.0 | 31.1 ± 5.6 |
|  | 20 g | 25.0 ± 3.0 | −0.9 ± 3.0 | 6.9 ± 4.4 | 2.6 ± 3.9 | 2.7 ± 4.5 | 1.1 ± 3.3 | 32.9 ± 5.1 | 28.0 ± 4.1 |
|  | 40 g | 25.0 ± 3.2 | 0.1 ± 2.4 | 3.5 ± 4.0 | 2.3 ± 3.5 | 5.9 ± 3.8 | 0.8 ± 3.7 | 28.0 ± 4.4 | 30.6 ± 4.7 |
| Tired | NB | 48.5 ± 4.2 | 2.0 ± 3.1 | −3.5 ± 4.3 | −6.8 ± 4.9 | 2.4 ± 4.1 | −2.8 ± 4.5 | 52.0 ± 4.4 | 52.2 ± 4.4 |
|  | 20 g | 54.0 ± 3.2 | −4.3 ± 3.3 | −5.0 ± 2.8 | −6.3 ± 3.9 | −3.4 ± 4.0 | −13.7 ± 4.9 | 45.6 ± 4.2 | 55.5 ± 4.3 |
|  | 40 g | 55.6 ± 3.1 | −2.8 ± 2.7 | −5.4 ± 3.4 | −6.3 ± 4.1 | −7.3 ± 4.4 | −9.7 ± 5.3 | 51.1 ± 4.5 | 49.9 ± 4.6 |
| Tense | NB | 27.7 ± 3.3 | 1.7 ± 3.3 | 3.1 ± 3.2 | −1.5 ± 2.9 | 1.4 ± 3.3 | −2.8 ± 2.8 | 31.8 ± 3.6 | 28.2 * **†** ± 3.1 |
|  | 20 g | 28.4 ± 3.5 | 0.2 ± 2.1 | −2.1 ± 3.4 | −0.8 ± 2.9 | 3.5 ± 3.5 | −3.8 ± 3.1 | 30.4 ± 3.8 | 37.2 ± 4.7 |
|  | 40 g | 30.1 ± 3.2 | −1.6 ± 2.7 | −2.8 ± 2.7 | −2.8 ± 3.9 | −2.3 ± 3.4 | −2.4 ± 3.4 | 28.9 ± 3.3 | 33.4 ± 3.5 |
| Headache | NB | 20.3 ± 3.7 | 1.7 ± 3.2 | 3.6 ± 3.3 | 0.5 ± 3.6 | 4.2 ± 4.5 | −1.7 ± 3.6 | 26.1 ± 4.0 | 19.3 ± 3.7 |
|  | 20 g | 18.3 ± 3.4 | −0.1 ± 2.1 | −1.0 ± 2.8 | −0.1 ± 3.2 | −1.2 ± 3.4 | −7.3 ± 2.6 | 17.2 ± 2.7 | 19.8 ± 3.6 |
|  | 40 g | 18.5 ± 3.2 | −1.0 ± 1.4 | −3.8 ± 3.0 | −0.4 ± 2.5 | −4.4 ± 2.7 | −6.5 ± 2.4 | 22.1 ± 4.2 | 22.6 ± 3.3 |
| Overall Mood | NB | 56.7 ± 3.5 | −2.3 ± 2.3 | 4.0 ± 2.1 | 0.3 ± 2.3 | −4.5 ± 2.6 | 10.6 ± 2.1 | 58.6 * ± 3.6 | 63.1 ± 3.7 |
|  | 20 g | 57.5 ± 2.7 | 3.3 ± 2.1 | 9.0 ± 2.1 | 3.7 ± 2.3 | 1.2 ± 3.0 | 12.0 ± 3.2 | 63.5 ± 3.3 | 58.8 ± 3.2 |
|  | 40 g | 56.3 ± 3.2 | 4.0 ± 2.3 | 7.0 ± 2.4 | 6.6 ± 2.9 | 2.7 ± 3.7 | 12.3 ± 3.4 | 64.9 ± 3.3 | 61.9 ± 3.3 |
| Mental Fatigue | NB | 33.3 ± 3.6 | 5.5 * ± 3.1 | 0.1 ± 4.1 | 4.8 ± 4.0 | 7.9 ± 3.7 | 0.3 ± 4.2 | 42.1 ± 4.6 | 39.3 ± 4.6 |
|  | 20 g | 36.8 ± 3.3 | 0.6 ± 2.8 | 0.5 ± 3.4 | 0.6 ± 4.7 | 4.1 ± 5.6 | −4.4 ± 5.2 | 39.5 ± 4.5 | 45.4 ± 4.3 |
|  | 40 g | 38.9 ± 4.4 | −6.2 ± 3.7 | −3.9 ± 3.5 | −2.7 ± 4.4 | −3.9 ± 4.8 | −7.8 ± 5.1 | 40.2 ± 4.6 | 38.1 ± 4.7 |
| Post-Cognitive VAS Mental Fatigue | NB | 44.8 ± 3.3 | 5.3 ± 2.9 | 4.0 ± 3.5 | 8.4 ± 3.2 | 10.3 ± 3.6 | 3.1 ± 3.1 | 47.8 ± 4.9 | 48.7 ± 4.9 |
|  | 20 g | 48.5 ± 3.3 | 1.4 ± 1.9 | 3.1 ± 3.8 | 4.9 ± 3.8 | 0.7 ± 5.2 | 2.4 ± 4.6 | 60.9 ± 4.2 | 62.8 ± 3.4 |
|  | 40 g | 49.2 ± 3.0 | −3.0 ± 3.6 | 1.0 ± 3.1 | −0.2 ± 3.6 | −2.2 ± 3.8 | −1.0 ± 3.7 | 50.6 ± 4.8 | 60.2 ± 4.0 |
| Difficulty | NB | 42.3 ± 3.5 | 3.3 ± 2.2 | 4.5 ± 3.1 | 6.1 ± 3.1 | 7.4 ± 3.4 | 7.3 ± 3.0 | 51.8 ± 4.1 | 48.7 ± 3.7 |
|  | 20 g | 44.4 ± 3.9 | 2.0 ± 2.9 | −1.0 ± 3.4 | 0.9 ± 4.2 | 2.3 ± 4.2 | 1.0 ± 4.0 | 56.7 ± 4.8 | 52.2 ± 3.7 |
|  | 40 g | 43.0 ± 3.1 | −1.5 ± 2.4 | −3.4 ± 3.5 | −3.7 ± 3.5 | −4.4 ± 3.4 | −4.3 ± 4.8 | 48.9 ± 3.6 | 49.4 ± 3.6 |

Means ± SEM are presented; * Mean value was significantly different from 40 g breakfast (*p* < 0.05); **†** Mean value was significantly different from 20 g breakfast (*p* < 0.05).

**Table S3.** Time-averaged AUC values for subjective appetite measures following no breakfast (NB) or a 20 g or 40 g breakfast before, during and after exercise.

| **Measure** | **Condition** | **AUC (mm)** | | | | **AUC (%)** |
| --- | --- | --- | --- | --- | --- | --- |
|  |  | **Baseline** | **Post-Breakfast** | **Pre-Post Exercise** | **Post-Exercise Recovery Period** | **Afternoon/Evening** |
| Hunger | NB | 61 ± 3 | 17 * **†** ± 3 | 17 * **†** ± 3 | 59 * **†** ± 3 | 24 ± 3 |
|  | 20 g | 61 ± 4 | 15 ± 3 | 15 * ± 3 | 54 * ± 2 | 24 ± 3 |
|  | 40 g | 59 ± 3 | 10 ± 2 | 12 ± 2 | 50 ± 2 | 24 ± 3 |
| Desire to eat | NB | 61 ± 3 | 13 * **†** ± 3 | 14 * **†** ± 3 | 61 * **†** ± 3 | 27 ± 2 |
|  | 20 g | 64 ± 2 | 11 * ± 2 | 14 * ± 3 | 58 * ± 1 | 29 ± 3 |
|  | 40 g | 58 ± 3 | 10 ± 2 | 14 ± 3 | 53 ± 2 | 27 ± 3 |
| Fullness | NB | 30 ± 3 | 16 * **†** ± 3 | 13 * **†** ± 3 | 32 * **†** ± 2 | 37 ± 4 |
|  | 20 g | 31 ± 4 | 13 ± 3 | 16 ± 3 | 36 * ± 2 | 39 ± 3 |
|  | 40 g | 30 ± 3 | 11 ± 2 | 14 ± 3 | 41 ± 2 | 39 ± 4 |
| Satisfaction | NB | 39 ± 3 | 16 * **†** ± 3 | 11 * **†** ± 2 | 36 * **†** ± 3 | 37 ± 3 |
|  | 20 g | 38 ± 4 | 14 ± 3 | 13 ± 3 | 43 * ± 2 | 38 ± 3 |
|  | 40 g | 35 ± 2 | 11 ± 2 | 14 ± 3 | 48 ± 2 | 38 ± 4 |

Mean values ± SEM are presented; * Mean value was significantly different from 40 g breakfast (*p* < 0.05); † Mean value was significantly different from 20 g breakfast (*p* < 0.05).

**Document S1.** Cognitive Task and VAS Descriptions.

Computerized Cognitive Tasks

Four Choice Reaction Time (FCRT; ~90 s)

Four direction arrow keys were displayed on the computer screen. The arrows “lit up” at irregular intervals (between 1 and 3.5 s), one at a time. Participants were instructed to use the index finger of their dominant hand to press the corresponding button on the response pad (left/right/up/down) when an arrow became lit, responding as quickly and as accurately as possible. In total, 32 stimuli were presented. The task was scored for RT and accuracy of responses.

Stroop Task (~2 min)

Words describing one of four colours (“RED”, “YELLOW”, “GREEN”, “BLUE”) were presented in different coloured fonts in the centre of the computer screen. The participant pressed one of four coloured response buttons in order to identify the font colour (e.g., if the word ‘GREEN’ was presented in a blue font, the correct response would be to respond with the blue button). The presented words were either “congruent” (word and font are the same colour) or “incongruent” (word and font are different colours) and were presented in a random order. In total, 120 words were presented. The task was scored for RT and accuracy of responses to “congruent” and “incongruent” words.

N-Back (~2 min)

A series of single letters appeared on the screen, presented one at a time. If the letter that appeared was also presented three letters previously in the series (a “target” letter), participants were asked to press the “YES” button on the response pad and if the letter that appeared was not presented three letters previously in the series (a “non-target” letter), to press the “NO” button on the response pad, responding as quickly and accurately as possible to every letter presented. In total, 36 stimuli were presented, encompassing 12 target pairs. The task was scored for RT and accuracy.

Rapid Visual Information Processing Task (RVIP; 5 min)

A series of single digit numbers between 1 and 9 appeared on the screen continuously at a rate of 100 per min. Participants were required to use the index finger of their dominant hand to press the centre button on the response box, reacting as quickly and accurately as possible, when they identified three odd or even digits presented in succession. Eight correct target strings were presented each minute. The task was scored for percentage of target strings correctly detected, average RT for correct detections and number of false alarms (incorrect responses).

Mobile Phone Cognitive Tasks

Arrow Reaction Time (ART; ~90 s)

An arrow pointing left or right appeared in the centre of the mobile phone screen at irregular intervals (between 1 and 2.5 s). The participant was instructed to press a key to identify the direction of the arrow (4 for “LEFT”, 6 for “RIGHT”), responding as quickly and as accurately as possible. The task was scored for overall RT and accuracy.

N-Back (~2 min)

A series of single letters appeared on the screen, presented one at a time. Participants were instructed to press a key to identify if the letter that appeared was presented 2 letters previously in the series (4 for “YES”, 6 for “NO”), responding as quickly and accurately as possible to every letter presented. The task was scored for RT and accuracy, both overall and to correct and incorrect and target and non-target responses separately.

Rapid Visual Information Processing Task (RVIP; 5 min)

A series of single digit numbers between 1 and 9 appeared on the screen continuously at a rate of 100 per min^−1^ for 5 min. Participants were required to use the index finger of their dominant hand to press the 4 button on the keypad, reacting as quickly and accurately as possible, when they identified three odd or even digits presented in succession. Eight correct target strings were presented each minute. The task was scored for percentage of target strings correctly detected, average RT for correct detections and number of false alarms (incorrect responses).

Computer Visual Analogue Scales

Mental Fatigue and Task Difficulty VAS

Two single visual analogue scales measuring mental fatigue and task difficulty were completed at the end of each set of tasks. Participants were asked to click on a 100 mm line on the computer screen to rate each variable. Each scale was labelled “not at all” (left end of scale) and “extremely” (right end of scale).

Mood and Physical State VAS

Participants were asked to click on a 100mm line on the computer screen to grade their current subjective status for each mood and physical state (“relaxed”, “alert”, “jittery”, “tired”, “tense”, “headache”, “overall mood”). Each scale was labelled “not at all” (left end of scale) and “extremely” (right end of scale), except for “overall mood” which was labelled “very bad” and “very good”. During the main study trials, participants also completed three further 100 mm VAS; immediately following breakfast and lunch to rate “liking” and immediately following the exercise session to rate the extent to which they enjoyed the run. All VAS scales were scored as mm along the line towards “extremely”.

Appetite VAS

Participants were asked to click on a 100 mm line on the computer screen (or draw a vertical line on a 100 mm line during exercise) to grade their current subjective status for feelings of “hunger”, “fullness”, “satisfaction”, “desire to eat”, “thirstiness”). Each scale was labelled “not at all” (or similar, left end of scale) and “extremely” (or similar, right end of scale).

Participants could not refer to their previous ratings when completing the VAS. All VAS scales were scored as mm along the line towards “extremely”.

Mobile Phone Visual Analogue Scales

The mobile phone VAS were identical to those described for the computer except participants were asked to move a cross left or right (using the 4 or 6 buttons on the keypad) on a 25 mm line on the mobile phone screen to grade their current subjective status for each state.

© 2015 by the authors; licensee MDPI, Basel, Switzerland. This article is an open access article distributed under the terms and conditions of the Creative Commons Attribution license (http://creativecommons.org/licenses/by/4.0/).
